# Diabetes promotes invasive pancreatic cancer by increasing systemic and tumour carbonyl stress in *Kras*^*G12D/+*^ mice

**DOI:** 10.1186/s13046-020-01665-0

**Published:** 2020-08-10

**Authors:** Stefano Menini, Carla Iacobini, Luisa de Latouliere, Isabella Manni, Martina Vitale, Emanuela Pilozzi, Carlo Pesce, Paola Cappello, Francesco Novelli, Giulia Piaggio, Giuseppe Pugliese

**Affiliations:** 1 Department of Clinical and Molecular Medicine, “La Sapienza” University, Via di Grottarossa, 1035-1039 -, 00189 Rome, Italy; 2 SAFU-unit, Department of Research, Advanced Diagnostics, and Technological Innovation, Regina Elena National Cancer Institute, Rome, Italy; 3 Pathology Unit, University “La Sapienza”, Sant’Andrea Hospital, Rome, Italy; 4 DINOGMI, University of Genoa Medical School, Genoa, Italy; 5 Department of Molecular Biotechnology and Health Sciences, University of Turin, Turin, Italy

**Keywords:** Pancreatic ductal adenocarcinoma, Hyperglycaemia, Reactive carbonyl species, Methylglyoxal, Advanced glycation end-products, Carnosine derivatives, Yes-associated protein, Large tumour suppressor kinase 1, Epidermal growth factor receptor, Extracellular signal-regulated kinases 1/2

## Abstract

**Background:**

Type 1 and 2 diabetes confer an increased risk of pancreatic cancer (PaC) of similar magnitude, suggesting a common mechanism. The recent finding that PaC incidence increases linearly with increasing fasting glucose levels supports a central role for hyperglycaemia, which is known to cause carbonyl stress and advanced glycation end-product (AGE) accumulation through increased glycolytic activity and non-enzymatic reactions. This study investigated the impact of hyperglycaemia on invasive tumour development and the underlying mechanisms involved.

**Methods:**

*Pdx1-Cre;LSL-Kras*^*G12D/+*^ mice were interbred with mitosis luciferase reporter mice, rendered diabetic with streptozotocin and treated or not with carnosinol (FL-926-16), a selective scavenger of reactive carbonyl species (RCS) and, as such, an inhibitor of AGE formation. Mice were monitored for tumour development by in vivo bioluminescence imaging. At the end of the study, pancreatic tissue was collected for histology/immunohistochemistry and molecular analyses. Mechanistic studies were performed in pancreatic ductal adenocarcinoma cell lines challenged with high glucose, glycolysis- and glycoxidation-derived RCS, their protein adducts AGEs and sera from diabetic patients.

**Results:**

Cumulative incidence of invasive PaC at 22 weeks of age was 75% in untreated diabetic vs 25% in FL-926-16-gtreated diabetic and 8.3% in non-diabetic mice. FL-926-16 treatment suppressed systemic and pancreatic carbonyl stress, extracellular signal-regulated kinases (ERK) 1/2 activation, and nuclear translocation of Yes-associated protein (YAP) in pancreas. In vitro, RCS scavenging and AGE elimination completely inhibited cell proliferation stimulated by high glucose, and YAP proved essential in mediating the effects of both glucose-derived RCS and their protein adducts AGEs. However, RCS and AGEs induced YAP activity through distinct pathways, causing reduction of Large Tumour Suppressor Kinase 1 and activation of the Epidermal Growth Factor Receptor/ERK signalling pathway, respectively.

**Conclusions:**

An RCS scavenger and AGE inhibitor prevented the accelerating effect of diabetes on PainINs progression to invasive PaC, showing that hyperglycaemia promotes PaC mainly through increased carbonyl stress. In vitro experiments demonstrated that both circulating RCS/AGEs and tumour cell-derived carbonyl stress generated by excess glucose metabolism induce proliferation by YAP activation, hence providing a molecular mechanism underlying the link between diabetes and PaC (and cancer in general).

## Background

Pancreatic cancer (PaC) is the tenth most common incident cancer, but the seventh leading cause of cancer-related death worldwide [1], because of the poor 5-year survival outcomes [2]. Due to the rising prevalence of risk factors such as obesity and type 2 diabetes, PaC is expected to become the second leading cause of cancer-related death in the US by 2030 [3]. Type 2 diabetes was found to be associated with a 5–7-fold higher risk of PaC in the first year after diabetes diagnosis and nearly twofold thereafter [4, 5]. Though type 2 diabetes is the main contributor to this problem, the entity and temporal trajectory of PaC risk were recently reported to be similar in type 1 diabetes [6], suggesting a common mechanism related to hyperglycaemia. This concept is supported by the recent finding that PaC incidence increases linearly with increasing fasting glucose levels, even within the normal range [7].

Previous studies have shown that type 2 diabetes induced by a high-fat diet promotes PaC [8, 9]. However, this experimental model of the metabolic syndrome does not allow assessing the role of hyperglycaemia independent of confounding factors such as obesity and hyperinsulinemia, thus hindering the understanding of the mechanisms underlying the risk conferred by hyperglycaemia. We have recently demonstrated that advanced glycation end-products (AGEs) promote proliferation of human pancreatic ductal adenocarcinoma (PDA) cell lines and that exogenous AGE administration markedly accelerates invasive tumour development in a mouse model of *Kras*-driven PaC [10]. Accumulation of AGEs in diabetes is mainly due to increased formation of reactive carbonyl species (RCS) derived from glucose auto-oxidation (e.g. glyoxal, GO), but also from cell metabolism of excess glucose through glycolysis (e.g., methylglyoxal, MGO) [11]. In turn, RCS react with amino groups of proteins causing structural and functional modifications. The resulting irreversible adducts (i.e., AGEs) accumulate in tissues, where they can exert further biological effects through interaction with specific receptors [12, 13].

Carnosine (beta-alanyl-L-histidine) is an endogenous histidine-containing dipeptide that inhibits AGE formation by scavenging RCS [14]. Though L-Carnosine was proven to be effective in several carbonyl stress-related disease conditions [15], including metabolic disorders [16–19], its therapeutic use in humans is hampered by the presence of high levels of serum carnosinase, thus prompting the search for carnosinase-resistant carnosine derivatives [18–20]. The novel bioavailable compound carnosinol, i.e., (2S)-2-(3-amino propanoylamino)-3-(1H-imidazol-5-yl) propanol (FL-926-16) [18–22], was shown to be highly effective in attenuating diabetes-associated vascular complications [21, 22] and obesity-related metabolic dysfunctions [18, 19]. Moreover, it was recently shown that L-carnosine is effective in counteracting glycolysis-dependent tumour growth by quenching RCS [23].

This study aimed at investigating whether hyperglycaemia associated with experimental type 1 diabetes favours the progression of preneoplastic lesions to malignancy in a well-validated mouse model of PaC by increasing carbonyl stress. To this end, mice were treated with the RCS scavenger and inhibitor of AGE formation FL-926-16. An additional objective was to analyse the effect of the diabetic *milieu* and of FL-926-16 on the activity of Yes-associated protein (YAP), a key downstream target of KRAS signalling required for progression of pancreatic intraepithelial neoplasias (PanINs) to invasive PaC [24, 25] and for MGO-induced tumour growth [23].

## Methods

### In vivo study

The experimental protocols comply with the principles of (https://www.nc3rs.org.uk/arrive-guidelines) and were approved by the National Ethics Committee for Animal Experimentation of the Italian Ministry of Health (Authorization no. 1470/2015-PR). The mice were housed in single cages with wood-derived bedding material in a specific pathogen-free facility with a 12-h light/dark cycle under controlled temperatures (20–22 °C). Mice were cared for in accordance with the Principles of Laboratory Animal Care (National Institutes of Health publ. no. 85–23, revised 1985) and with national laws, and received water and food ad libitum. The primary and secondary endpoint were the development of invasive PaC and the development/progression of PanINs, respectively.

#### Design

The effect of diabetes on PaC progression was investigated in *Pdx1-Cre;LSL-Kras*^*G12D/+*^ (KC) mice, which develop autochthonous PaC in a pattern recapitulating human pathology with high fidelity by developing the full spectrum of PaC progression, from preneoplastic lesions (PanINs) to adenocarcinoma and metastasis [26, 27]. KC mice were interbred with mitosis luciferase (*MITO–Luc*) reporter mice to obtain KC-Mito (KCM) mice [10, 28, 29]. The *LSL-Kras*^*G12D/+*^ lineage was maintained in the heterozygous state. Mice were screened by polymerase chain reaction (PCR) using tail DNA amplified by specific primers to the Lox-P cassette flanking mutated *Kras*^*G12D/+*^, wild type *Kras*, *Cre* recombinase and *MITO* genes, as previously reported [10, 29]. In the *MITO-Luc* mouse, an artificial minimal promoter derived from the cyclin B2 gene and induced by NF-Y drives the expression of the luciferase reporter specifically in replicating cells. Therefore, both normal (e.g., bone marrow) and tumour actively proliferating cells may be localized by a bioluminescence imaging (BLI)-based screen [10, 28, 29]. We have previously shown that KCM mice develop pre-invasive (PanINs) and invasive ductal PaC with the same penetrance, latency, and histological features as those described for KC mice [29]. According to the Ethics Committee recommendations, to limit the number of animals, the experiments were stopped when it was sufficient to confirm or reject the working hypothesis in a statistically and clinically meaningful manner.

**Fig. 1:**
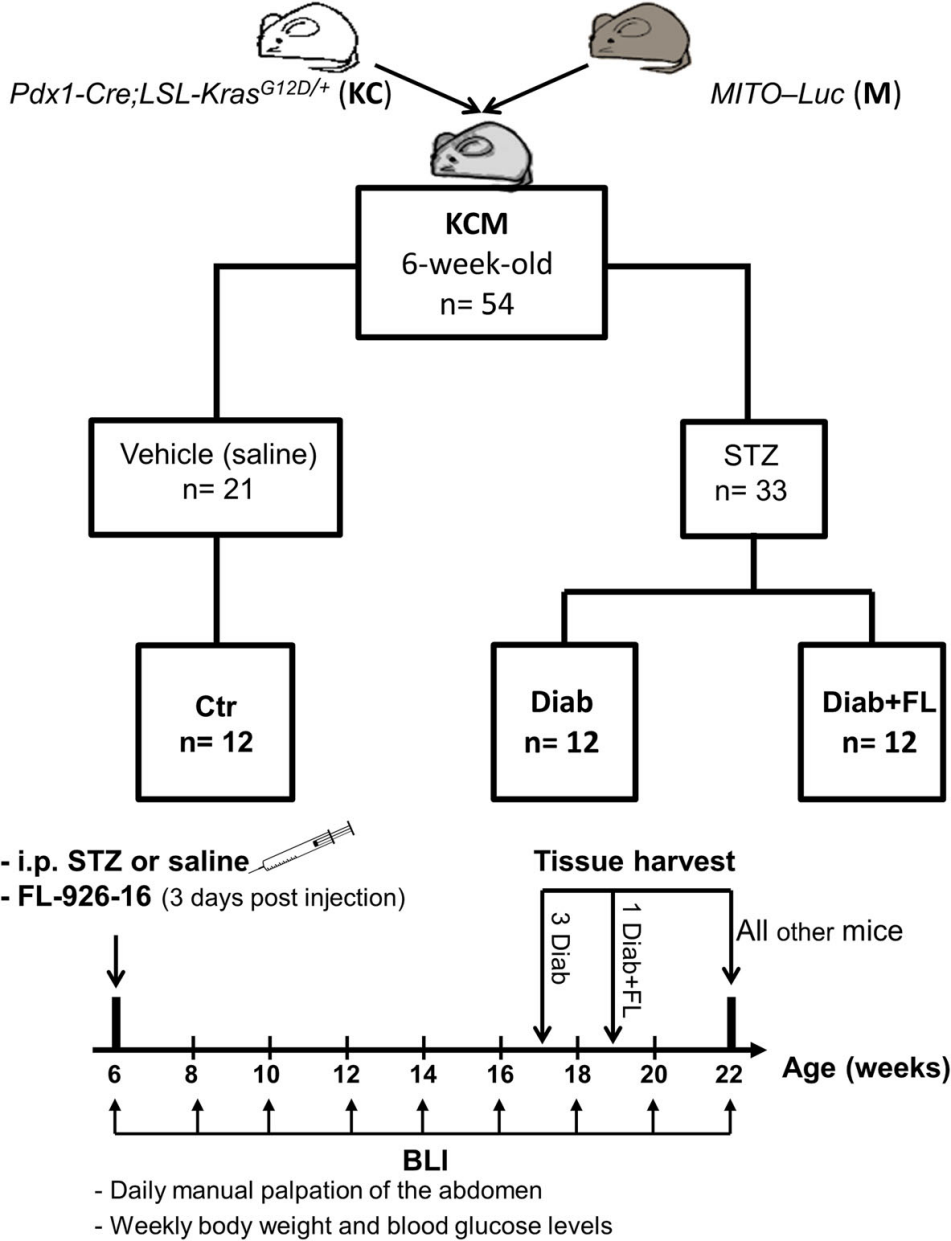
Flowchart and timeline of study design. Please refer to the text for detailed description. In dashed boxes, groups of non-diabetic KCM mice (Ctr) that served as control for the effect of STZ (STZ-non-Diab) or FL-926-16 (FL) treatment (Ctr + FL) on PaC development and progression. To avoid unnecessary suffering, three diabetic mice (Diab) and one Diab mouse treated with FL (Diab+FL) were killed 5 and 3 weeks, respectively, before the end of the study. STZ = streptozotrocin; BLI = bioluminescence imaging

Figure 1 shows the flowchart and timeline of study design. Thirty-three KCM mice were rendered diabetic with streptozotocin (STZ) and followed for 16 weeks (i.e., up to 22 weeks of age). After an overnight fast, 6-week-old mice were intraperitoneally injected with 190 mg·kg^− 1^ STZ (Sigma-Aldrich, St. Louis, MO, USA). Success rate, defined as the percentage of STZ-injected mice with glucose levels > 250 mg/dL for the entire study period was 72.7% (24/33). Three days after injection, diabetic mice were randomized to receive no treatment (Diab, *n* = 12) or FL-926-16 (gift of Flamma S.p.A., Chignolo d’Isola, Italy) [30] at a dose of 30 mg·kg^− 1^∙day^− 1^ in the drinking water (Diab+FL, n = 12) and injected weekly with 1 IU of insulin glargine to prevent excessive weight loss and ketoacidosis. FL-926-16 was shown to have a suitable absorption, distribution, metabolism, excretion, and toxicity (ADMET) profile, and the greatest potency and selectivity toward RCS among all other carnosine derivatives [18]. The FL-926-16 dose was chosen based on previous results from our group [22], showing high efficacy in preventing diabetes-induced renal injury, and from other investigators, indicating a good safety profile at the dose of 10–45 g·kg^− 1^·day^− 1^ [18, 30]. Neither histological abnormalities of the liver, kidney, lung, and heart, nor functional abnormalities attributable to toxicity on these tissues were observed in this study or in a previous one [22]. STZ-treated mice not fulfilling the criteria for diabetes diagnosis (STZ-non-Diab, *n* = 9) served as control for STZ effect on PaC; seven of these mice failed to develop hyperglycaemia, whereas two had spontaneous recovery from diabetes within 2 weeks. Vehicle (saline)-injected KCM mice were used as non-diabetic controls and either left untreated (Ctr; *n* = 12) or treated with FL-926-16 (Ctr + FL; n = 9) to check for any drug effect.

Mice were subjected to in vivo BLI every other week [10, 29] and daily manual palpation of the abdomen to check for tumour growth and avoid the loss of animals, along with the need to cope with the related ethical issues (i.e., compliance with the 3Rs principles). Briefly, 10 min after administration of D-luciferin (75 mg/kg body weight, intraperitoneal; Perkin Elmer, Hopkinton, MA, USA), photon emission from the different body areas was acquired for 5 min and analysed with a CCD camera (Xenogen IVIS Lumina System; Perkin Elmer). A specific region of interest (ROI) corresponding to the abdominal area occupied by the pancreas was manually selected and the total photon flux (p/s) from this ROI was evaluated with Living Image software (Caliper Life Sciences; Perkin Elmer) [10, 28, 29].

At the end of the study, mice were anaesthetized with ketamine (60 mg·kg^− 1^ Imalgene i.p.) and xylazine (7.5 mg·kg^− 1^ Rompum i.p.) and killed by cervical dislocation. According to the Ethics Committee recommendations, to avoid suffering, three Diab and one Diab+FL mice presenting with both positive BLI and a palpable abdominal mass or poor general condition were killed 5 and 3 weeks, respectively, before the end of the study. The lungs and the middle part of the gastrointestinal tract, including the pancreas and the liver, were dissected, and exposed to the CCD camera for 5 min for photon emission assessment. The pancreas was dissected, photographed and weighted; then one part was stored at − 80 °C for molecular analysis, whereas the other part was processed for histological/immunohistochemical analysis [10]. At time of collection, a technician (C.C., see Acknowledgements) recoded biological samples to allow blinded analysis.

#### Metabolic parameters

Body weight and blood glucose were monitored weekly. At the end of the study, the levels of haemoglobin (Hb) A1c, an indicator of long-term glycaemic control, were assessed by using the Mouse HbA1c Assay Kit (80,310, Crystal Chem, Zaandam, Netherlands), and serum AGEs and total protein carbonyls (PCOs), two carbonyl stress markers, were measured by ELISA (OxiSelect™ Advanced Glycation End-Product Competitive ELISA Kit, no. STA-817 and OxiSelect™ Protein Carbonyl ELISA Kit, no. STA-310, respectively, Cell Biolabs, Inc., San Diego, CA, USA).

#### Pancreas histology

Six 4-μm-thick non-serial pancreatic sections stained with haematoxylin and eosin were examined to confirm the presence of invasive PaC. Pancreas without invasive PaC were analysed to grade dysplastic ducts (i.e., PanINs) according to previously established criteria [26]. The numbers of low-grade (PanIN-1A/B) and high-grade (PanIN-2/3) dysplastic ducts were counted and expressed as a percentage of total ducts in the specimen [10].

#### Pancreatic AGEs

*ERK 1/2 phosphrylation status, nuclear YAP, and its target gene connective tissue growth factor (CTGF).* Levels of AGEs, p-ERK 1/2, and CTGF protein in homogenates and of active (non-phosphorylated) YAP1 in nuclear extracts of pancreas of mice were assessed by Western blot. Human PDA tissues (*n* = 14) were obtained from the Pathology Unit of Sant’Andrea Hospital, Rome, Italy, in agreement with the ethical guidelines established by the locally appointed Ethics Committee. Pancreatic tissue distribution of AGEs and activated YAP1 in mouse and human specimens were evaluated by dual label immunofluorescence and immunoperoxidase, respectively [10, 31]. For immunofluorescence, a goat polyclonal anti-AGE antibody and a rabbit monoclonal antibody to active (non-phosphorylated) YAP1 were used as primary antibodies, followed by appropriate secondary fluorescent antibodies (see Supplementary Table S1 for antibodies in Additional file 1). Sections were analysed at a fluorescence microscope (Zeiss AXIO A1), equipped with an Axiocam 503 color camera (Carl Zeiss Italy, Milan, Italy). For immunoperoxidase, formalin-fixed paraffin embedded sections (4-μm thick) were rehydrated and treated with 0.3% H_2_O_2_ in PBS for 30 min to block endogenous peroxidase activity. Heat mediated antigen retrieval was performed with “Antigen Unmasking Solution, Citric Acid Based” (H-3300, Vector Laboratories, Burlingame, CA, USA) for AGE staining, or Tris/EDTA buffer pH 9.0, for YAP staining, both for 20 min. Nonspecific binding was blocked by incubation in Protein block serum free (Agilent/Dako, Santa Clara, CA, USA) for 30 min at room temperature. Then, sections were incubated with Avidin/Biotin blocking Kit (SP-2002, Vector Laboratories) for 30 min, an anti-AGE antibody (Abcam, Cambridge, UK, ab23722) or an antibody directed to the active (non-phosphorylated) YAP1 (Abcam, ab205270) at 4 °C overnight, and the appropriate biotinylated secondary antibody at room temperature for 30 min (see Supplementary Table S1 for antibodies in Additional file 1). Finally, sections were stained with UltraTek Horseradish Peroxidase (ABL015, ScyTek Laboratories, UT, USA) for 10 min followed by 3,3-diaminobenzidine (DAB)/H_2_O_2_ Chromogen/Substrate Kit (High Contrast) (ACV500, ScyTek Laboratories) until the reaction product was visualized (~ 3 min), and counterstained with hematoxylin. AGE positive staining and nuclear expression of YAP were measured in 10 random fields of each section at a final magnification of 250X and 400X, respectively, by means of the interactive image analyzer Image-Pro Premier 9.2 (Immagini&Computer, Milan, Italy). AGE positivity was expressed as the mean percentage of field’s area occupied by the specific stain. Expression status of active YAP in tumor specimens was assessed by counting the number of nuclei positive for YAP and expressed as the mean ratio (%) of YAP-positive nuclei to total nuclei.

### In vitro study

The in vitro study investigated the putative role of RCS and AGEs as mediators of the tumour-promoting effect of high glucose (HG) and the protective effect of the carbonyl-sequestering agent and AGE inhibitor FL-926-16.

#### Design

Human MIA PaCa-2 (Catalogue No. 85062806, Lot No. 14A02) and Panc-1 (Catalogue No. 87092802, Lot No. 10G011) cells (Sigma-Aldrich) were used for assessing the effects of HG and FL-926-16 on cell proliferation. Experiments aimed at investigating the molecular mechanisms underlying the glucose-mediated effects and the protection by FL-926-16 were conducted on MIA PaCa-2 cells. Mycoplasma contamination in cell cultures was regularly tested by PCR MycoSPY Kit (Biontex Laboratories GmbH, Munchen, Germany). Human PDA cells were maintained in *DMEM* supplemented with 10% FBS and incubated in different conditions for three days, i.e., (1) normoglycaemia (normal glucose, 5 mM); (2) hyperglycaemia (HG, 25 mM); treated with (3) MGO or GO (200 μM, Sigma-Aldrich), two RCS and AGE precursors, or (4) the preformed AGE N^ε^-carboxymethyllysine (CML, 100 μg/mL), prepared as previously reported [10, 21], with or without FL-926-16 (20 mM); and (5) exposed to *DMEM* low glucose medium containing 10% of pooled sera from non-diabetic or diabetic individuals, before and after AGE removal from diabetic serum by an immunoadsorption method (see below), with or without FL-926-16 (20 mM). Informed consent was obtained from non-diabetic and diabetic individuals. Moreover, both YAP and Epidermal Growth Factor Receptor (EGFR) were silenced to assess the role of YAP and EGFR pathway in RCS and AGE-induced cell proliferation (see below).

#### Removal of AGEs from diabetic serum

AGEs were removed from diabetic serum using an immunoadsorption method. To immunoprecipitate AGE-modified proteins, 500 μl of diabetic serum was incubated for 1 h with 25 μl of Pierce NHS-activated magnetic beads (Thermofisher Scientific) covalently conjugated with 10 μg of anti-AGE antibody (Abcam, see Supplementary Table S1 for antibodies in Additional file 1), according to the manufacturer instruction. To confirm the efficiency of AGE depletion, AGE concentration in both treated (unbound serum fraction) and untreated diabetic serum was evaluated in triplicate by ELISA (OxiSelect™ Advanced Glycation End-Product Competitive ELISA Kit, no. STA-817, Cell Biolabs, Inc., San Diego, CA, USA). Following this procedure, the concentration of AGEs in diabetic serum was reduced by about 60%, reaching a concentration similar to that of the non-diabetic serum (see the “Results” section).

#### YAP and EGFR silencing

YAP and EGFR were silenced using small interfering RNAs (siRNAs) and irrelevant scrambled siRNAs as control (Thermo Fisher Scientific, Waltham, MA, USA). Validated predesigned siRNA oligonucleotides and related TaqMan assays are detailed in Supplementary Table S2 (see Additional file 1). Lipofectamine RNAiMAX (Thermo Fisher Scientific) transfections were performed using 20 nM of each siRNA.

#### Cell survival and proliferation

Cell viability and proliferation were evaluated by Cytoselect WST-1 Cell Proliferation Assay (Cell Biolabs) following the manufacturer instructions.

*YAP1, its upstream regulators large tumour suppressor Kinase 1(LATS1) and EGFR-ERK pathway, and its molecular targets CTGF, WTN5A and EMP2 in in human PDA cells.* Cells were extracted in 1% SDS buffer containing protease and phosphatase inhibitors (Sigma Aldrich). Nuclear protein extracts were obtained from cell monolayers with the Nuclear Extract Kit (Active Motif Corp., Carlsbad, CA, USA). Protein concentrations were determined using the Bradford Assay Kit (Bio-Rad Hercules, CA, USA). Nuclear protein levels of YAP1 and cellular protein levels of total and EGFR phosphorylated at Tyr1068 (p-EGFR), total and p-ERK 1/2 and LATS1, a key kinase of the Hippo pathway [32], were assessed by Western blotting (see Supplementary Table S1 for antibodies in Additional file 1). KRAS activity was evaluated by the KRAS activation Assay Kit (no. STA-400-K Cell Biolabs, Inc.) according to the manufacturer’s protocol. Briefly, 1 mg of lysate was subjected to pull-down and 50 μg of lysate was used to measure total KRAS. Pull-down and total lysates were subjected to Western blotting procedure using the primary antibody against KRAS provided by the kit. The mRNA levels of *CTGF/CCN2*, *WTN5A and EMP2*, three recognized molecular targets of YAP [23, 33, 34], were assessed by real-time PCR (RT-PCR) using a StepOne Real-Time PCR System and TaqMan Gene Expression assays (Thermo Fisher Scientific) [10] listed in Supplementary Table S3 (see Additional file 1).

### Statistical analysis

Results are expressed as mean ± SD, mean ± SEM or percentage. Differences between cell types/treatments or animal groups were assessed by one-way ANOVA followed by the Student-Newman-Keuls test for multiple comparisons, or two-way ANOVA followed by the Bonferroni post-test, as appropriate. Between-group differences in PaC incidence were assessed using the Chi-squared test and Fisher’s exact test to compute a *P*-value from a contingency table. A *P*-value of < 0.05 was considered to be significant. All statistical tests, including linear regression analysis, were performed on raw data using GraphPad Prism version 5.00 for Windows (GraphPad Software, San Diego, CA, USA).

## Results

### In vivo study

#### Metabolic parameters

**Fig. 2:**
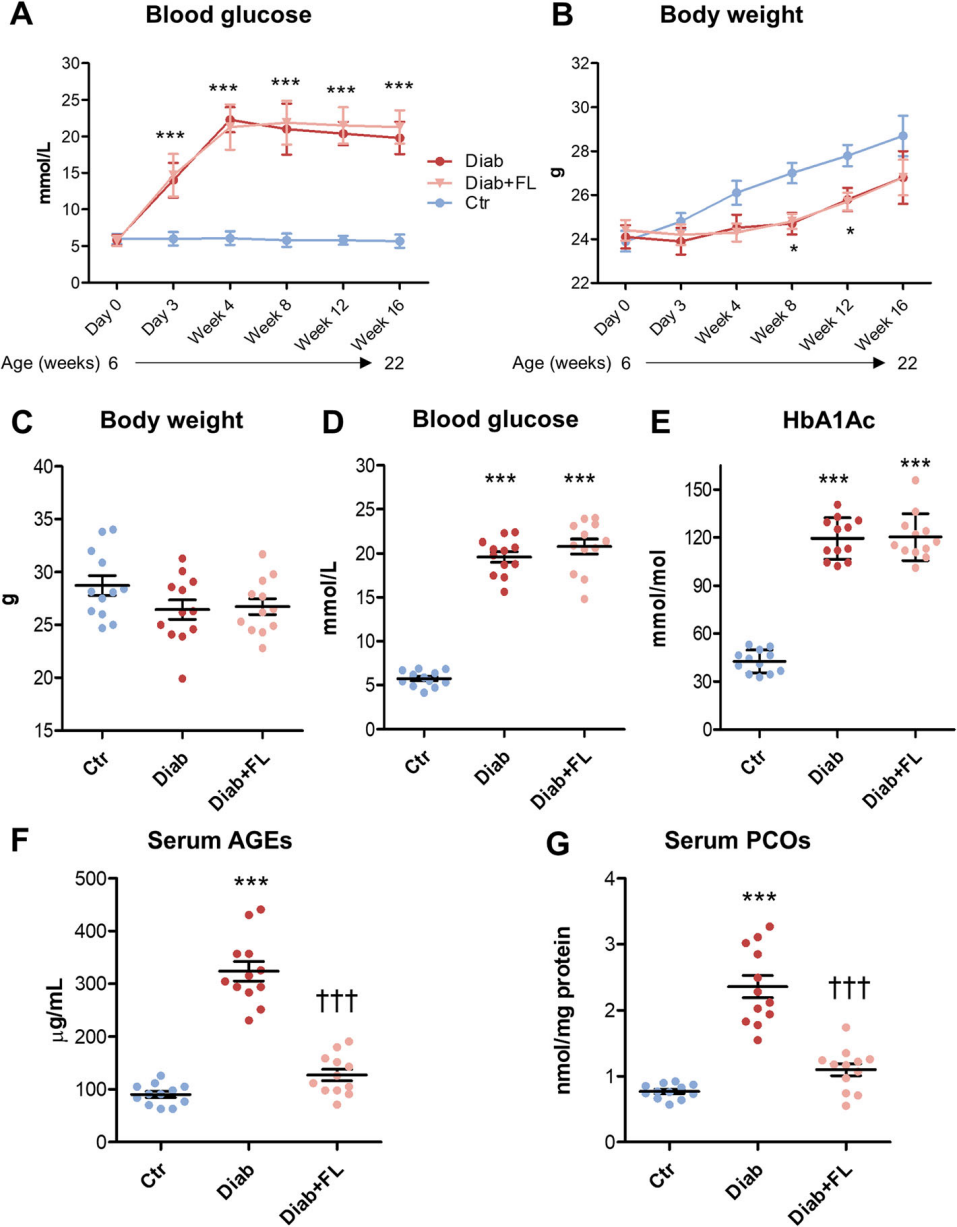
Glucose and HbA1c levels, body weight and hyperglycaemia-associated carbonyl stress. Blood glucose levels and body weight during the study period (**a** and **b**) and at the end of the study period (22 weeks of age^***1***^) (**c** and **d**) and HbA1c levels (**e**), and serum levels of AGEs (**f**) and total PCOs (**g**) at the end of the study period (22 weeks of age^***1***^) in control (Ctr), Ctr treated with FL-926-16 (Ctr + FL), diabetic (Diab), and Diab treated with FL-926-16 (Diab+FL) KCM mice. Statistical significance between groups for time course of blood glucose (**a**) and body weight (**c**) was calculated using two-way ANOVA followed by the Bonferroni post-test. Each time point represents mean ± SD of 12 animals until the 17th week of age, and 9–12 animals from the 18th to the 22nd week of age. Statistical significance for blood glucose (**c**), body weight (**d**), serum levels of AGEs (**e**) and PCOs (**f**) at 22 weeks of age^***1***^ was assessed using one-way ANOVA followed by the Student-Newman-Keuls test for multiple comparisons. Each dot represents one case and bars represent mean ± SEM. ***P < 0.001 or *P < 0.05 vs Ctr; †††*P* < 0.001 vs Diab. ^***1***^*Except for three Diab and one Diab + FL mice, which were killed 5 and 3 weeks, respectively, before the end of the study (see “Results” section for further details)*.

STZ-treated KCM mice developed hyperglycaemia starting about 72 h post-injection (Fig. 2a) and showed a slight decline in the growth curve vs Ctr mice, which reached statistical significance only at 8 and 12 weeks of age (Fig. 2b). Despite no difference in body weight (Fig. 2c), blood glucose (Fig. 2d), and HbA1c levels (Fig. 2e), FL-926-16 treatment prevented the diabetes-associated increase in circulating AGEs (Fig. 2f) and total PCOs (Fig. 2g), as assessed at the end of the study.

##### Invasive PaC development

**Fig. 3:**
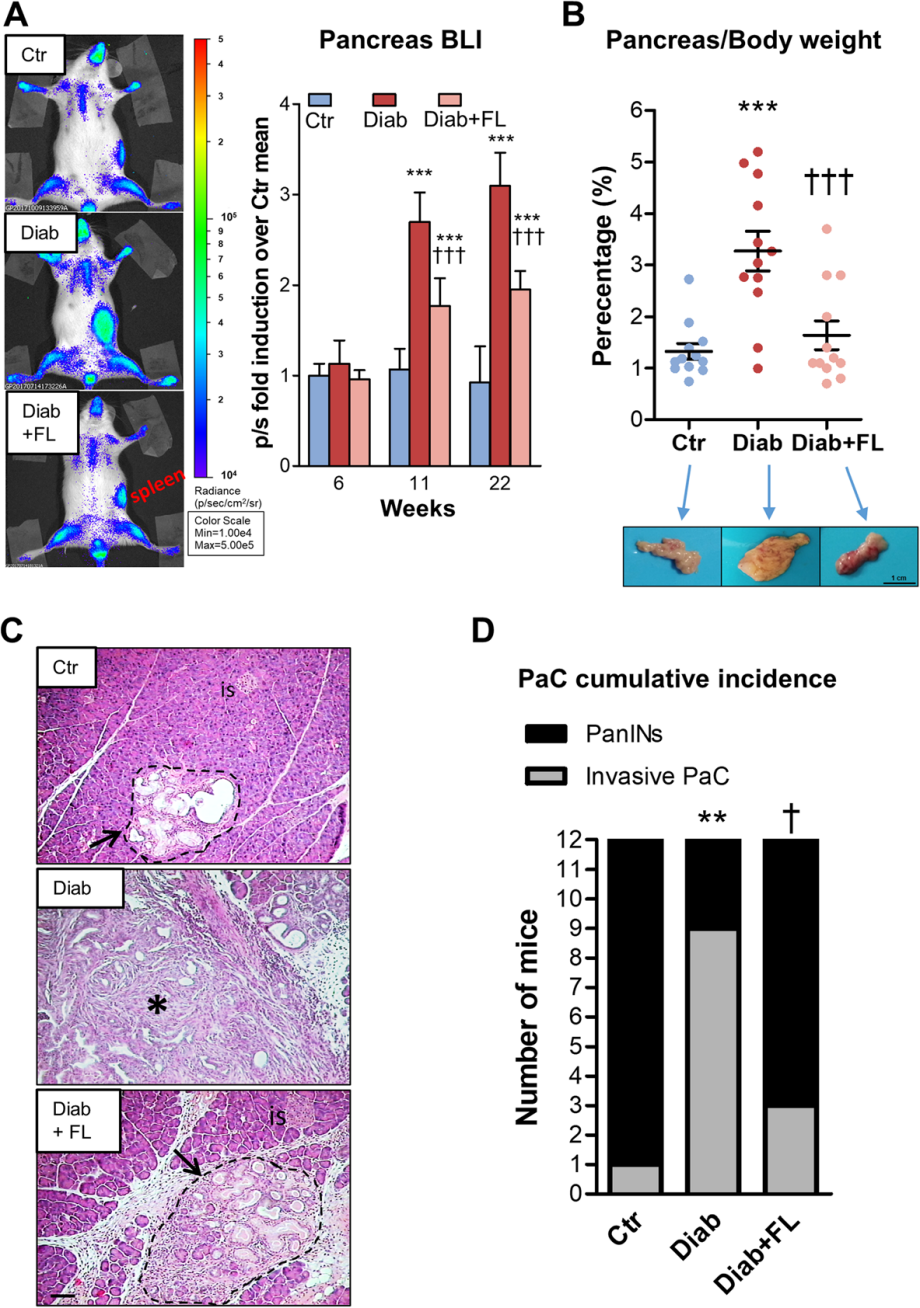
In vivo BLI and gross and microscopic examination of pancreas. Representative BLI at the end of the study period and total photon flux (p/s) induction from pancreas at 6, 11 and 22 weeks of age^***1***^ (**a**), pancreas/body weight percent ratio (**b**), representative pancreas histology (**c**, original magnification: 100X, scale bar: 200 μm), and cumulative incidence of PaC (**d**) in control (Ctr), diabetic (Diab), and Diab treated with FL-926-16 (Diab+FL) KCM mice at the time of sacrifice. Statistical significance between groups for pancreas/body weight percent ratio (**a**) was calculated using one-way ANOVA followed by the Student-Newman-Keuls test for multiple comparisons. Each dot represents one case and bars represent mean ± SEM. Statistical significance for PaC incidence (**b**) was assessed using the Chi-squared test and Fisher’s exact test. **P < 0.01 vs Ctr; †*P* < 0.05 vs Diab. Is = islet, * = invasive PaC, arrows = PanINs. ^***1***^*Except for three Diab and one Diab + FL mice, which were killed 5 and 3 weeks, respectively, before the end of the study (see “Results” section for further details)*.

Representative BLI images at the end of the study period and total photon flux induction from pancreas at 6, 11, and 22 weeks of age are shown in Fig. 3a. At sacrifice, pancreas weight was significantly (*P* < 0.01) increased in Diab (0.82 ± 0.29 g) vs Ctr (0.38 ± 0.14 g) and vs Diab+FL (0.44 ± 0.27 g) KCM mice. Pancreas/body weight percent ratio was almost tripled in Diab vs Ctr mice, whereas no statistical difference was observed between Diab+FL and Ctr mice (Fig. 3b and Table 1). As assessed by histology (Fig. 3c), cumulative incidence of invasive PaC at 22 weeks of age was 75% in Diab mice vs 25% in Diab-FL and 8.3% in Ctr mice (Fig. 3d and Table 1). Representative BLI images and pancreas histology from Ctr, Diab and Diab+FL are shown in Fig. 3c-d. Neither the Ctr + FL nor the STZ-non-Diab group showed significant differences in the incidence invasive PaC and pancreas/body weight percent ratio vs the Ctr group (Table 1). Furthermore, no between-group differences were observed in tumour invasiveness, except for an apparent reduction in Diab+FL vs Diab group (Table 1). However, the few cases of PaC in Diab+FL (*n* = 3) and Ctr (*n* = 1) mice prevent to perform statistical comparisons among groups for metastatic disease. Representative ex vivo BLI and histology images of liver and lung metastases are presented in Supplementary Fig. S1 in Additional file 1. Grading of dysplastic ducts in mice free of invasive PaC (Table 2) showed significant differences between Diab+FL and Diab mice for the percentage of normal ducts, which was higher, and of high-grade PanINs, which was lower, in the FL-926-16- treated arm. In addition, Ctr + FL mice presented with higher normal ducts and lower low-grade PanINs vs Ctr mice, whereas no difference was observed between STZ-non-Diab and Ctr mice.

**Table 1:** Pancreatic cancer (PaC) incidence, Pancreas/Body weight (Wt) percent ratio and metastasis

	PaC N/tot	Pancreas/Body Wt%	Metastasis N/tot PaC
**Ctr**	1/12	1.3 ± 0.5	1/1
**Diab**^**1**^	9/12	3.3 ± 1.3***	8/9
**Diab + FL**^**1**^	3/12	1.6 ± 1.0†††	1/3
**Ctr + FL**	1/9	1.4 ± 0.5	1/1
**STZ-non-Diab**	1/9	1.3 ± 0.4	0/1

Cumulative incidence of PaC and Pancreas/Body weight (Wt) percent ratio in control (Ctr), diabetic (Diab), Diab treated with FL-926-16 (Diab+FL), Ctr treated with FL-926-16 (Ctr + FL) and streptozotocin-treated non-diabetic (STZ-non-Diab) KCM mice at the end of the study (16 weeks of diabetes, 22 weeks of age^***1***^). The number of KCM mice with metastasis (liver and or lung) on the total number of PaC cases is also shown. KCM = *LSL-Kras*^*G12D/+*^*; Pdx-1-Cre*; *MITO*; N/tot = number of cases/total number of mice; N/tot PaC = number of cases/total number of PaC. PaC (ductal adenocarcinoma) and hepatic and/or lung metastasis were confirmed by histology. ***P < 0.001 or **P < 0.01 vs Ctr; †††*P* < 0.001 or †*P* < 0.05 vs Diab. Statistical significance between groups for Pancreas/Body Weight percent ratio was calculated using one-way ANOVA followed by the Student-Newman-Keuls test for multiple comparisons. Statistical significance for PaC rate was assessed using the Chi-squared test and Fisher’s exact test

^1^ Except for three Diab and one Diab + FL mice, which were killed 5 and 3 weeks, respectively, before the end of the study

**Table 2:** Pancreatic intraepithelial neoplasia (PanIN) grading

	Normal ducts %	Low-grade PanINs %	High-grade PanINs %
**Ctr** (*n* = 11)	68.0 ± 7.3	26.6 ± 5.4	5.4 ± 5.6
**Diab** (n = 3)	31.7 ± 10.5 **	42.0 ± 6.6 **	26.3 ± 14.22 **
**Diab + FL** (n = 9)	51.3 ± 5.9 ** ††	37.4 ± 7.3 **	11.2 ± 4.3 ††
**Ctr + FL** (*n* = 8)	77.0 ± 6.2 * ††	20.4 ± 5.6 * ††	2.6 ± 1.5 ††
**STZ-non-Diab** (n = 8)	61.6 ± 9.7 ††	32.4 ± 7.4	6.6 ± 5.2 ††

PanIN grading in control (Ctr), diabetic (Diab), Diab treated with FL-926-16 (Diab+FL), Ctr treated with FL-926-16 (Ctr + FL) and streptozotocin-treated non-diabetic (STZ-non-Diab) KCM mice free of invasive pancreatic cancer as attested by histology at the end of the study (16 weeks of diabetes, 22 weeks of age). KCM = *LSL-Kras*^*G12D/+*^*; Pdx-1-Cre*; *MITO*. **P < 0.01 and *P < 0.05 vs Ctr; ††*P* < 0.01 vs Diab. Statistical significance between groups for Normal ducts, Low-grade (PanIN-1A/B) and High-grade (PanIN-2/3) dysplastic ducts was calculated using one-way ANOVA followed by the Student-Newman-Keuls test for multiple comparisons

##### Pancreatic AGEs

**Fig. 4:**
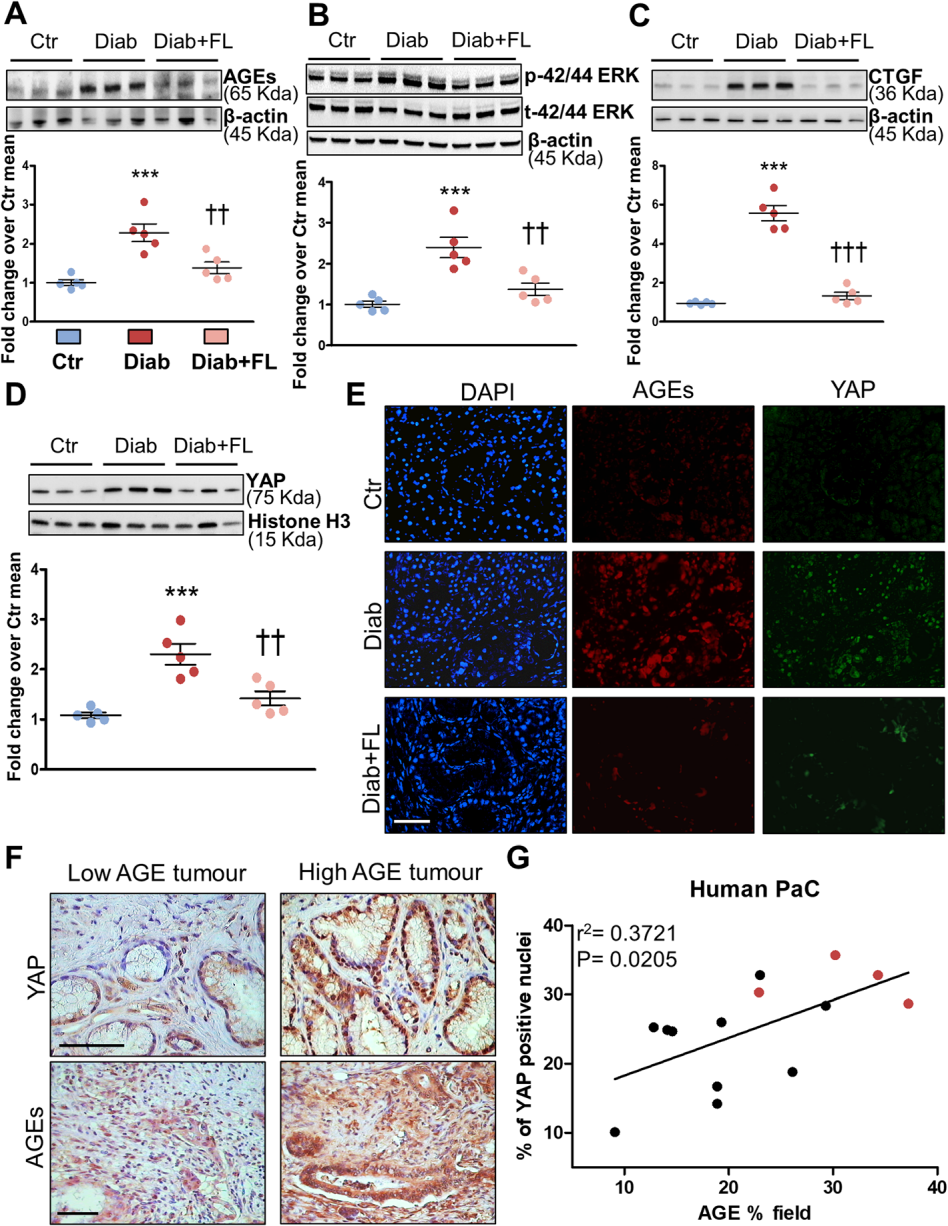
Association between AGE levels (carbonyl stress burden), ERK 1/2 phosphorilation status and YAP activation in murine and human PaC. Representative Western blots for AGEs (**a**), phosphorylated and total ERK 1/2 (**b**), CTGF (**c**) and nuclear YAP (**d**) in control (Ctr), diabetic (Diab), and Diab treated with FL-926-16 (Diab+FL) KCM mice at the time of sacrifice and relative band densitometry analysis from five mice per group. Each dot represents one case and bars represent mean ± SEM. Dual-label immunofluorescence (**d**) for AGEs (red) and YAP (green). DAPI (blue): 4′,6-diamidino-2-phenylindole. Original magnification: 250X, scale bar: 200 μm. Active (non-phosphorylated) YAP immunohistochemistry staining (**e**, upper panels, original magnification: 400X, scale bar: 200 μm) in representative low and high carbonyl stress human pancreatic adenocarcinomas as assessed by their AGE level (**e**, lower panels, original magnification: 250X, scale bar: 200 μm). Linear regression analysis (**f**) of the correlation between the ratio of YAP-positive nuclei to total nuclei with percentage of tumour tissue positive for AGE staining (*n* = 14). Red dots = patients with a clinical diagnosis of diabetes mellitus prior to undergoing surgery; black dots = non-diabetic subjects. Post hoc multiple comparison: ***P < 0.001 vs Ctr; †††*P* < 0.001 or ††*P* < 0.01 vs Diab

*ERK 1/2 phosphrylation status, nuclear YAP, and connective tissue growth factor (CTGF).* Pancreatic accumulation of AGEs (Fig. 4a) and levels p-ERK 1/2 (Fig. 4b), CTGF (Fig. 4c), a well-established transcriptional target of YAP [24, 33, 34], and nuclear YAP1 (Fig. 4d) were increased in Diab vs Ctr mice and increments were prevented by FL-926-16 treatment. Dual label immunofluorescence analysis confirmed the association between AGEs and nuclear YAP1 in PaC lesions from Diab mice (Fig. 4e). A significant positive relationship between AGE accumulation and nuclear YAP1 levels was also observed in human PDA (Fig. 4f-g).

### In vitro study

#### Proliferation of human PDA cells

**Fig. 5:**
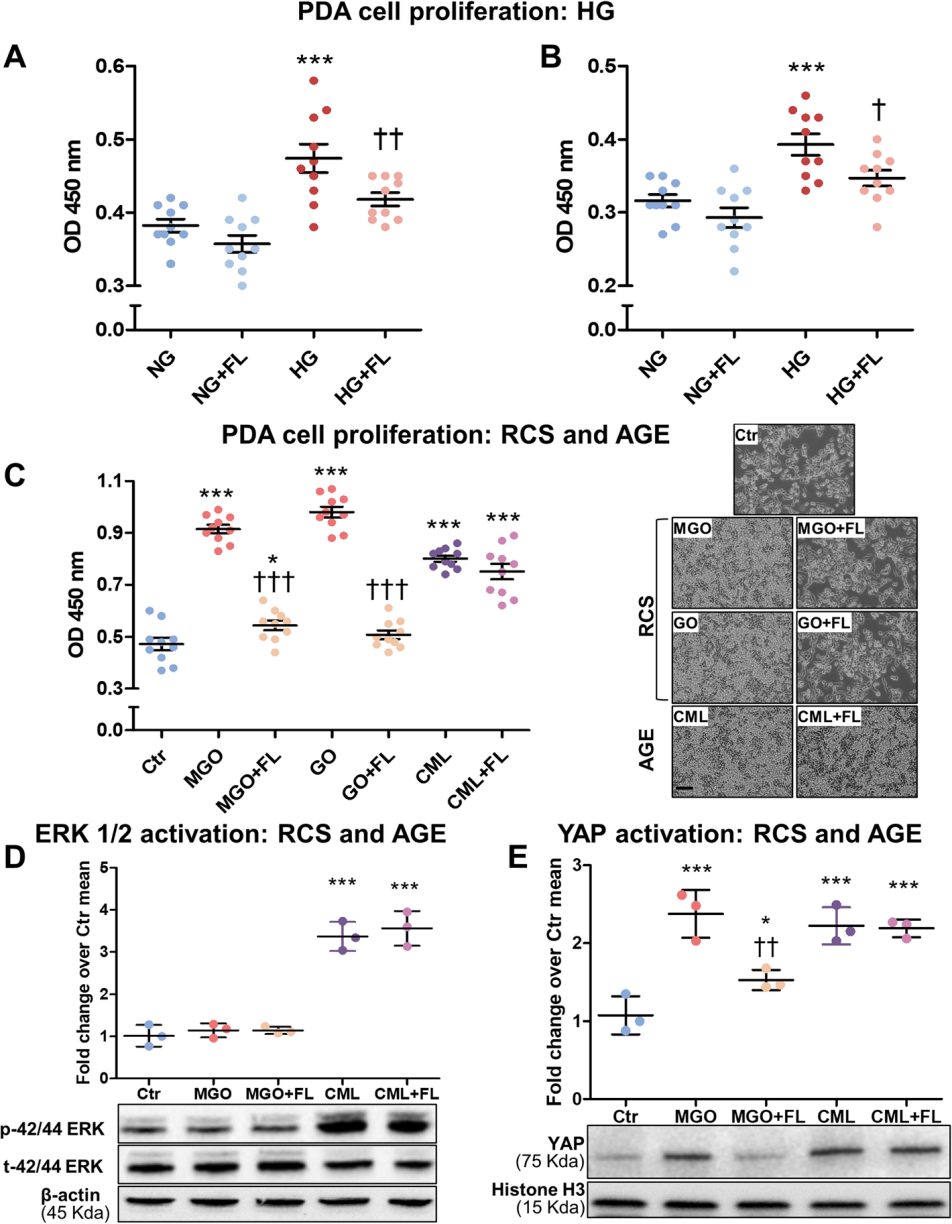
Effect of HG, RCS, AGE and FL-926-16 on human PDA cell proliferation, ERK 1/2 phosphorylation status and YAP activation. MIA PaCa-2 (**a**) and Panc-1 (**b**) cell proliferation after 48 h incubation with high glucose (HG, 25 mM) vs normal glucose (NG, 5 mM), with or without the carbonyl trapping agent FL-926-16 (FL, 20 mM). PDA cell (MIA PaCa-2, **c**) proliferation after 48 h incubation with the RCS glyoxal (GO) and methylglyoxal (MGO), 200 μM each, or the preformed AGE CML (100 μg/mL), with or without 20 mM FL, and relative representative bright-field images (original magnification: 100X, scale bar: 200 μm); *n* = 5 wells in duplicate per condition. Western blot analysis for phosphorylated and total ERK 1/2 in lysates (**d**) and YAP in nuclear extracts (**e**) from MIA PaCa-2 cells exposed to MGO (200 μM) or CML (100 μg/mL) for 48 h, with or without FL (20 mM), and relative band densitometry analysis from three separate experiments. Each dot in (**a**), (**b**) and (**c**), represents one well and bars represent mean ± SEM. Each dot in (**d**) and (**e**) represents a single experiment and bars represent mean ± SEM. Post hoc multiple comparison: ***P < 0.001 or *P < 0.05 vs Ctr; †††*P* < 0.001, ††*P* < 0.01 or †*P* < 0.05 vs untreated

HG concentration mimicking diabetic hyperglycaemia promoted PDA cell growth and this effect was prevented by FL-926-16 (Fig. 5a-b). The AGE precursors RCS, MGO and GO, and the preformed AGE CML also stimulated PDA cell proliferation. FL-926-16 was able to inhibit cell proliferation induced by MGO and GO, but not CML (Fig. 5c). Treatment with CML, but not with MGO, induced ERK 1/2 activation, and FL-926-16 was ineffective in counteracting the effect of CML on ERK 1/2 phosphorylation status (Fig. 5d). However, the proliferating effect of both the RCS MGO and the AGE CML was associated with YAP1 nuclear persistence and activity. Again, FL-926-16 efficiently prevented the nuclear translocation of YAP1 induced by MGO, but failed to counteract the effect of CML (Fig. 5e). Consistently, FL-926-16 treatment reversed the MGO-induced upregulation of gene expression of *CTGF*, Wnt Family Member 5A (*WNT-5A*) and Epithelial Membrane Protein 2 (*EMP2*), three well-recognized YAP target genes [24, 33, 34]. Conversely, FL-926-16 was ineffective in preventing the modulatory effect of CML on the mRNA level of these genes (Supplementary Fig. S2 in Additional file 1).

#### Mechanisms underlying RCS- and AGE-induced YAP activation

**Fig. 6:**
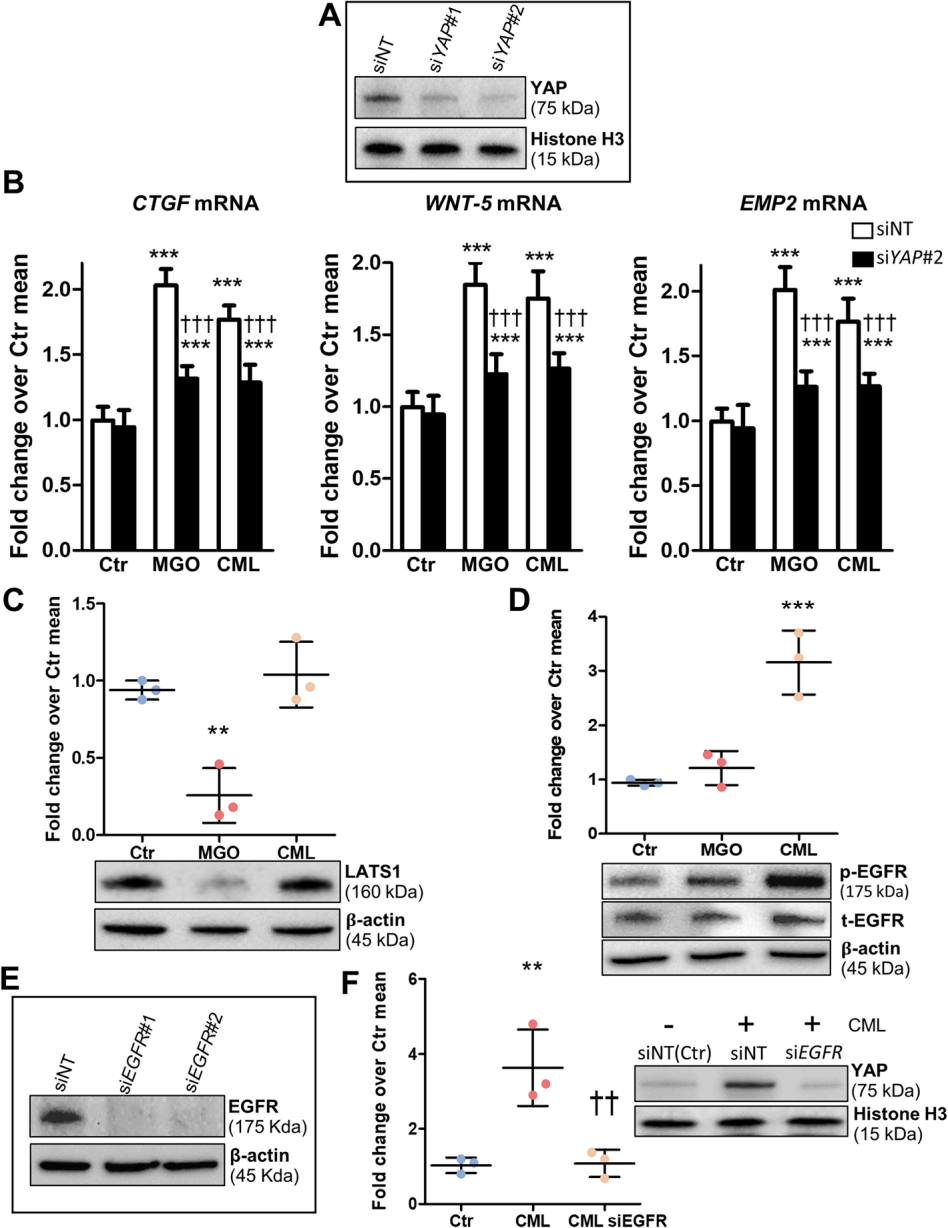
Differential mechanisms underlying RCS- and AGE-induced YAP activation. YAP protein level (**a**) by Western blot in MIA PaCa-2 siNT control and siYAP#1 and #2 cells after 48 h. *CTGF*, *WNT5a* and *EMP2* mRNA levels (**b**) in MIA PaCa-2 siNT control and siYAP#2 cells exposed to MGO (200 μM) or CML (100 μg/mL); n = 5 wells in duplicate per condition. LATS1 (**c**) and total and phosphorylated EGFR (**d**) levels in MIA PaCa-2 cells exposed to MGO (200 μM) or CML (100 μg/mL) for 48 h and relative band densitometry analysis from three separate experiments. Analysis of EGFR silencing by Western blot (**e**) in MIA PaCa-2 cells after 48 h. Western blot analysis for YAP (**f**) in nuclear extracts from MIA PaCa-2 siNT control and siEGFR cells exposed to CML (100 μg/mL) for 48 h and relative band densitometry analysis from three separate experiments. Each dot represents a single experiment and bars represent mean ± SD. Post hoc multiple comparison: ***P < 0.001, **P < 0.01 or *P < 0.05 vs Ctr; †††*P* < 0.001 or ††*P* < 0.01 vs treated (MGO or CML) siNT control. siNT = non-target control

Silencing of YAP1 using two independent siRNAs (si*YAP*#1 and #2) (Fig. 6a) significantly inhibited the transcription activity of YAP target genes induced by both MGO and CML in PDA cells (Fig. 6b). In MGO-treated cells, YAP induction was associated with a decrease in protein levels of LATS1, a well-established negative regulator of YAP activity [32], whereas CML treatment failed to modulate LATS1 (Fig. 6c). Instead, treatment with CML, but not with MGO, was found to induce EGFR phosphorylation (pEGFR) (Fig. 6d). EGFR silencing (Fig. 6e) almost completely reversed YAP1 nuclear translocation (Fig. 6f), KRAS activation, and ERK 1/2 phosphorylation (Supplementary Fig. S3A-B) induced by CML.

#### Effects of serum from diabetic patients on proliferation of human PDA cells

**Fig. 7:**
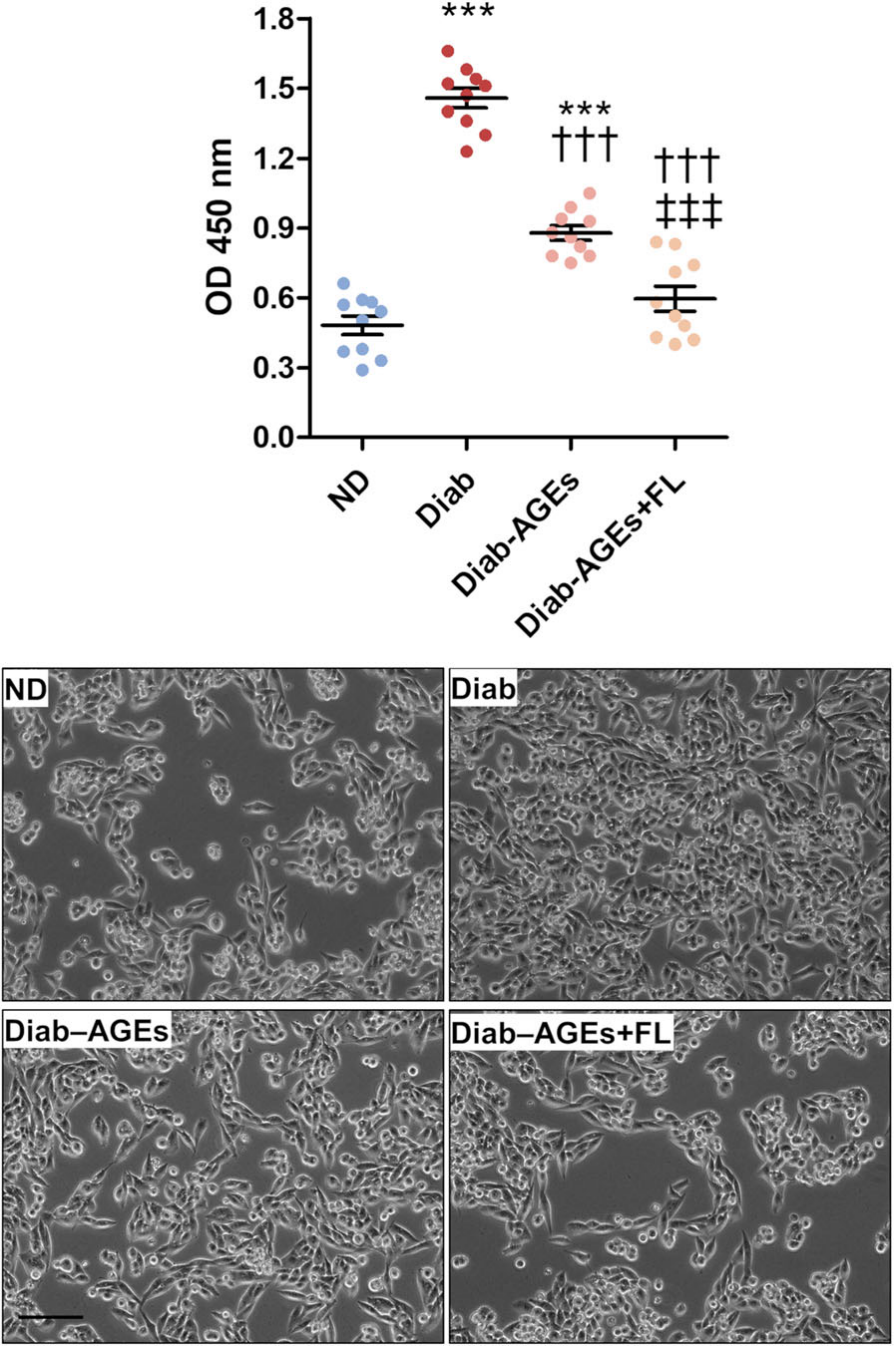
Effect of diabetic serum on human PDA cell proliferation. Mia PaCa-2 cell proliferation after 48 h incubation with medium containing 10% of serum from healthy, non-diabetic (ND) subjects or diabetic patients before (Diab) and after AGE removal in the absence (Diab-AGEs) or presence (Diab-AGEs+FL) of FL-926-16 (20 mM), and relative representative bright-field images (original magnification: 100X, scale bar: 200 μm); n = 5 wells in duplicate per condition. Each dot represents one well and bars represent mean ± SEM. Post hoc multiple comparison: ***P < 0.001 vs ND; †††*P* < 0.001 vs Diab; ‡‡‡*P* < 0.001 vs Diab-AGEs

The levels of AGEs were 178.2 ± 13.2 μg/mL in the pooled sera from diabetic patients and 72.3 ± 4.54 μg/mL in pooled sera from non-diabetic individuals. The diabetic serum induced a 3-fold increase in PDA cell proliferation compared to the non-diabetic serum. This effect was greatly reduced by prior selective AGE removal from the diabetic serum (AGE levels, 95.5 ± 7.92 μg/mL) and almost completely reversed by combining AGE removal from serum and FL-926-16 treatment of PDA cells (Fig. 7).

## Discussion

Despite the epidemiological evidence of increased PaC risk in both type 1 [6] and 2 [4, 5] diabetes, the underlying mechanisms still remains to be elucidated. Here we showed that STZ-induced type 1 diabetes, which is characterized by marked hyperglycaemia and insulinopenia without weight gain [35], significantly accelerated tumour progression in a mouse model of *Kras*–driven PaC. The absence of obesity and insulin resistance argues in favour of the hypothesis that the PaC-promoting effect of diabetes is directly related to the adverse effects of hyperglycaemia. In addition, RCS trapping and AGE inhibition by FL-926-16 efficiently prevented the acceleration of PanIN progression to invasive PaC induced by diabetes. The difference in the incidence of PaC between the two diabetic groups (i.e., untreated and treated with FL-926-16) occurred despite similar increases of blood glucose levels, supporting the concept that glucose metabolites, but not glucose per se, were responsible for PaC promotion. STZ-treated mice that failed to develop or reversed hyperglycaemia showed the same PaC incidence as the Ctr group, thus ruling out an effect of STZ on invasive PaC development in Diab mice.

**Fig. 8:**
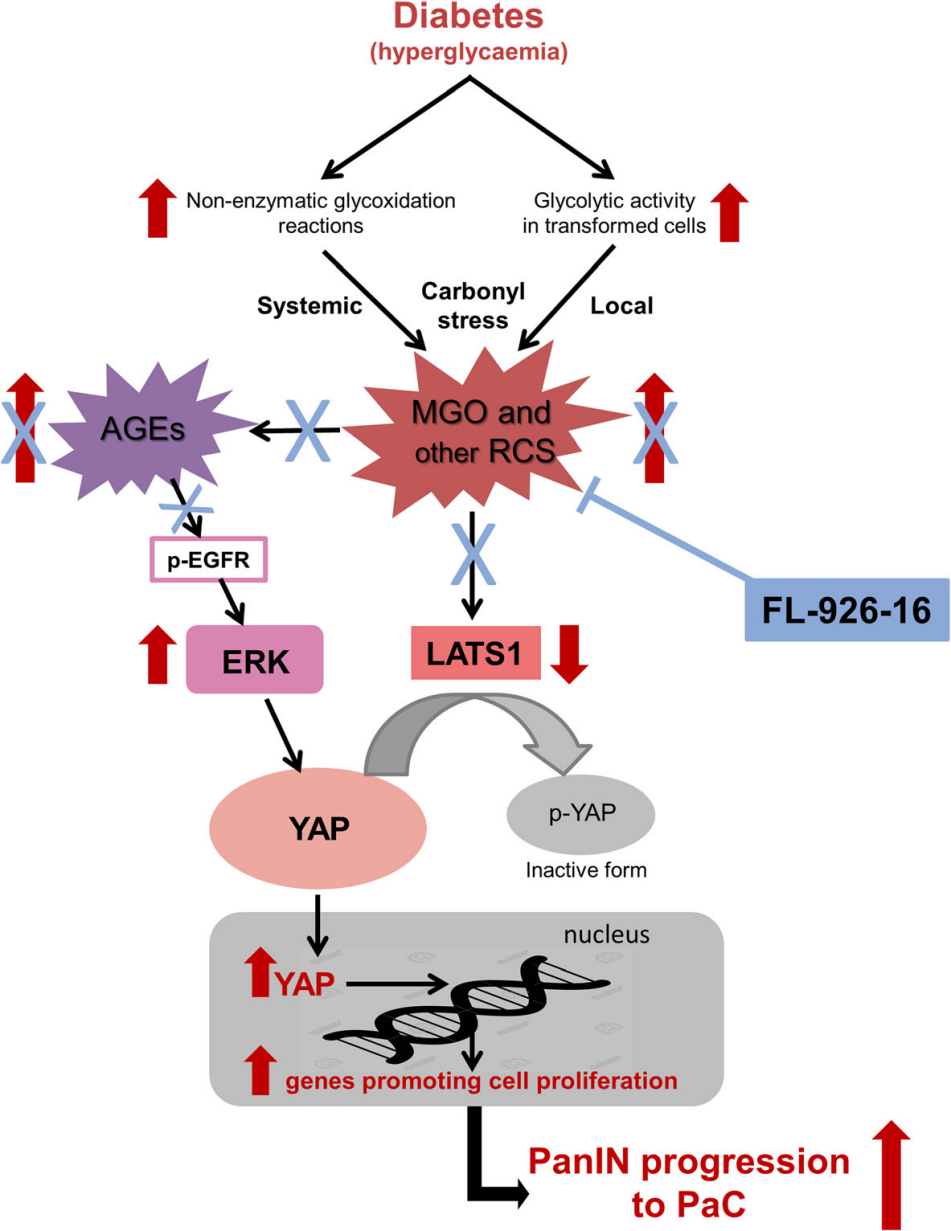
Carbonyl stress in PaC promotion induced by diabetes and mechanism of FL-926-16-mediated protection. Diabetes markedly accelerates tumour progression through hyperglycaemia-derived carbonyl stress. The increased availability of glucose feeds the glycolytic flux of tumour cells favouring local formation of RCS such as MGO, an inevitable side-product of glycolysis, and consequent AGE accumulation. In addition, circulating RCS and their protein adducts (i.e., AGEs) derived from non-enzymatic glycoxidation reactions occurring at the systemic level may also contribute to AGE accumulation in neoplastic lesions, which is associated with increased nuclear translocation of YAP, a key effector of Hippo pathway and regulator of tumour growth and invasion [24, 25, 34, 37]. Quenching of RCS and inhibition of AGE formation by the RCS sequestering agent FL-926-16 efficiently prevents hyperglycaemia-induced YAP activation and acceleration of PanIN progression to invasive PaC. LATS1 = large tumour suppressor kinase 1; ERK = extracellular signal-regulated kinase; p-EGFR = phosphorylated epidermal growth factor receptor; p-YAP = phosphorylated YAP

Our finding of an association between AGE accumulation and YAP induction in PaC in Diab mice is in line with previous evidence of a role of carbonyl stress in breast cancer [23, 36]. In this tumour type, a high content of RCS protein adducts (i.e., AGEs) was found to be associated with increased nuclear levels of the transcriptional co-activator YAP [23, 36], a key regulator of tumour growth and invasion [24, 25, 33, 37]. This finding is consistent with the observation that cellular glucose metabolism stimulates YAP activity [38, 39] through the production of the glycolysis side-product MGO [39]. Hence, from a mechanistic perspective, the high glycolytic rates of cancer cells would favour formation of MGO and other RCS, since they are inevitably generated as by-products of glycolysis [11, 23, 40, 41]. In turn, RCS and their protein adducts (i.e., AGEs) would promote YAP nuclear localization and activity. It was proposed that variability in carbonyl stress burden among different tumour subtypes may partly depend on differences in enzymatic detoxification rates of MGO [36]. However, enhanced carbonyl stress burden and consequent tumour accumulation of AGEs in diabetes may depend mainly on the accelerated production rate of RCS favoured by the unrestricted availability of glucose to glycolysis-dependent cancer cells. In addition, the increased systemic carbonyl stress and circulating RCS/AGE levels characterizing diabetes [42, 43] may also contribute to the progression of preneoplastic lesions (Fig. 8), as also indicated by our previous study [10].

We demonstrated that AGE accumulation is associated with higher nuclear YAP levels and activity also in human PaC specimens. These results, together with the finding that treatment with FL-926-16 was able to counteract the diabetes promoting effect on invasive PaC development, support the hypothesis that hyperglycaemia-induced carbonyl stress is a risk factor for increased incidence of PaC in the diabetic population, in line with the recent epidemiological observation that the risk of PaC increases linearly with increasing fasting glucose levels [7].

In vitro experiments provided further mechanistic insight into the role of carbonyl stress in diabetes-induced PaC promotion. First, the ability of diabetic serum to induce human PDA cell growth was significantly inhibited by prior selective AGE removal, confirming our previous observation that circulating AGEs can exert a promoting effect on tumour progression [10]. The residual proliferating effect of diabetic serum after AGE removal was attributable to serum factors favouring RCS formation (e.g., high glucose) in cancer cells, as indicated by the additional protection provided by FL-926-16, and supported by the finding that FL-926-16 prevented HG-induced PDA cell growth. Consistently, both preformed AGEs and their RCS precursors stimulated PDA cell proliferation, YAP nuclear translocation, and upregulation of its target genes. FL-926-16 treatment was effective in preventing cell proliferation and YAP activation induced by HG and RCS but, as expected, failed to provide protection when cells were incubated with preformed AGEs. Overall, these in vitro results are in agreement with the RCS-scavenging action of FL-926-16 [15–19, 21] and indicate that the cell growth effect induced by HG is mediated by both RCS and AGEs.

The role of YAP as a mediator of the effects exerted by RCS and AGEs was attested by the silencing experiments targeting this transcription regulator, which was previously shown to be critical in progression to PaC [24] and in MGO-induced breast tumour growth [23]. However, while the AGE precursors RCS were found to reduce Large Tumour Suppressor Kinase 1 levels, a negative regulator of YAP activity, the effect of AGEs on YAP induction was completely dependent on EGFR/ERK signalling (Fig. 8). The results of the EGFR silencing experiments are in line with previous studies showing the ability of AGEs to transactivate the EGFR [44, 45], the role of EGFR ligands in sustaining YAP activity, and the association of EGFR ligands with shorter overall survival in human PaC [33, 46]. The observation that AGEs induce EGFR phosphorylation in PDA cells takes on pathological relevance, as *Kras*-driven PaC growth was shown to be dependent on EGFR signalling [47, 48], and combined inhibition of EGFR and C-RAF led to complete regression of PaC [49]. Finally, the effect of MGO on LATS1 is in keeping with the previous finding that MGO favours proteasome degradation of this tumour suppressor [23].

The main strength of this study is the use of a type 1 diabetes model, which allowed us to evaluate the effect of hyperglycaemia on invasive PaC development independently of other confounders while accounting for the effect of STZ per se by including the STZ-non-Diab group. A possible limitation is the use of immunological instead of analytical techniques for assessing AGE levels. However, this approach allowed us to evaluate the overall AGE burden (i.e., carbonyl stress) and the tissue distribution of these by-products, whereas the effect of individual RCS and AGE structures were investigated in the in vitro experiments.

## Conclusions

This study shows that diabetes-associated hyperglycemia promotes PanIN progression to invasive PaC. FL-926-16, a selective RCS scavenger and AGE inhibitor, prevented the accelerating effect of diabetes on PanINs progression to invasive PaC and PDA cell proliferation, providing evidence of the involvement of carbonyl stress in the association between diabetes and increased risk of PaC. Overall, our study proposes a general molecular mechanism underlying the diabetes-cancer link and suggests that carnosinase-resistant carnosine derivatives represent a promising class of RCS-scavenging agents that might be useful not only in the treatment of metabolic disorders [18, 19] and their complications [21, 22], but also in risk management and prevention of cancer, particularly in high-risk diabetic individuals.

## Supplementary information

**Additional file 1.** Supplementary Table S1. Antibodies used in Western blot and IHC studies. Supplementary Table S2. Silencer select Validated/Predesigned siRNAs and related TaqMan assays. Supplementary Table S3. TaqMan Gene Expression assays. Supplementary Fig. S1. Color digital photo (A), ex vivo BLI (B) and histological analysis (C) of the lung (left) and the liver (right) of a Diab KCM mice with metastatic PaC. Supplementary Fig. S2. Effect of RCS, AGE and FL-926-16 on YAP activity. Supplementary Fig. S3. Effect of EGFR silencing on KRAS activity and p-ERK 1/2 levels in human PDA cells exposed to CML.

## Data Availability

The datasets used and/or analysed during the current study are available from the corresponding author upon reasonable request.

## Abbreviations

AGEs: Advanced glycation end-products
BLI: Bioluminescence imaging
CML: N^ε^-carboxymethyllysine
*CTGF*: Connective tissue growth factor gene
Ctr: Non-diabetic control mice
Ctr + FL: Non-diabetic control mice treated with FL-926-16
Diab: Diabetic mice
Diab+FL: Diabetic mice treated with FL-926-16
EGFR: Epidermal growth factor receptor
*EMP2*: Epithelial membrane protein 2 gene
ERK: Extracellular signal-regulated kinase
FL-926-16: (2S)-2-(3-amino propanoylamino)-3-(1H-imidazol-5-yl) propanol (carnosinol)
GO: Glyoxal
HG: High glucose
KC: *Pdx1-Cre;LSL-Kras*^*G12D/+*^
KCM: KC-*Mito*
MGO: Methylglyoxal
LATS1: large tumour suppressor Kinase 1
*MITO-Luc*: Mitosis luciferase
PaC: Pancreatic cancer
PanIN: Pancreatic intraepithelial neoplasia
PCOs: Total protein carbonyls
PCR: Polymerase chain reaction
PDA: Pancreatic ductal adenocarcinoma
p-EGFR: Phosphorylated-EGFR
p-ERK: Phosphorylated extracellular signal-regulated kinase
RCS: Reactive carbonyl species
RT-PCR: Real-time PCR
siRNA: Small interfering RNAs
STZ: Streptozotocin
STZ-non-Diab: Streptozotocin-injected non-diabetic mice
*WNT-5A*: Wnt family member 5A gene
YAP: Yes-associated protein.
