# Supplementary material for: Diabetes promotes invasive pancreatic cancer by increasing systemic and tumour carbonyl stress in KrasG12D/+ mice
Source: J Exp Clin Cancer Res. 2020 Aug 10;39:152. doi: 10.1186/s13046-020-01665-0 (PMC7418209; doi:10.1186/s13046-020-01665-0)
Supplement: Supplementary file 1 — Additional file 1. Supplementary Table S1. Antibodies used in Western blot and IHC studies. Supplementary Table S2. Silencer select Validated/Predesigned siRNAs and related TaqMan assays. Supplementary Table S3. TaqMan Gene Expression assays. Supplementary Fig. S1. Color digital photo (A), ex vivo BLI (B) and histological analysis (C) of the lung (left) and the liver (right) of a Diab KCM mice with metastatic PaC. Supplementary Fig. S2. Effect of RCS, AGE and FL-926-16 on YAP activity. Supplementary Fig. S3. Effect of EGFR silencing on KRAS activity and p-ERK 1/2 levels in human PDA cells exposed to CML. [file 13046_2020_1665_MOESM1_ESM.pdf]

Supplementary Tables

Supplementary Table S1. Antibodies used in Western blot and IHC studies.

Target	Antibody	Catalog Nr.	Supplier
**Primary**			
AGEs (WB & IHC/IP)	Rabbit polyclonal	ab23722	Abcam, Cambridge, UK
AGEs (IHC/IF)	Goat polyclonal	DPBT-66761GA	Creative Diagnostics, Shirley, NY, USA
total ERK 1/2 (WB)	Rabbit momoclonal	4695	Cell Signaling Technology Leiden, The Netherlands
phospho ERK 1/2 (WB)	Rabbit monoclonal	4376	Cell Signaling Technology Leiden, The Netherlands
CTGF (WB)	Rabbit polyclonal	ab6992	Abcam, Cambridge, UK
active YAP1 (WB & IHC/IP-IF)	Rabbit monoclonal [EPR19812]	ab205270	Abcam, Cambridge, UK
LATS1 (WB)	Rabbit polyclonal	ab70561	Abcam, Cambridge, UK
pEGFR (WB)	Rabbit polyclonal	2234	Cell Signaling Technology, Leiden, The Netherlands
tot EGFR (WB)	Rabbit polyclonal	2232	Cell Signaling Technology, Leiden, The Netherlands
**Secondary**			
AGEs (WB), p-ERK 1/2/tot ERK 1/2, CTGF, active YAP1 (WB), LATS1, p-EGFR/tot EGFR	HRP-conjugated goat anti-rabbit	P0448	Agilent/Dako, Santa Clara, CA, USA
AGEs (IHC/IP), active YAP1 (IHC/IP)	Ultra Teck Anti-polyvalent Biotinylated Anti-IgG	ABN0015	ScyTek Laboratories, Utah, USA
AGEs (IHC/IF)	Alexa Fluor® 594 chicken anti-goat IgG	A-21468	Thermo Fisher Scientific, Waltham, MA, USA
active YAP1 (IHC/IF)	DyLight®488 goat anti-rabbit IgG	35552	Thermo Fisher Scientific, Waltham, MA, USA

AGEs = advanced glycation end-products; WB = Western blot; IHC = immunohistochemistry; IP = immunoperoxidase; IF = immunofluorescence; p-ERK 1/2 = phosphorylated Extracellular signal-regulated kinases, CTGF = connective tissue growth factor; YAP1 = yes associated protein 1; LATS1 = large tumor suppressor kinase 1; p-EGFR = phosphorylated epidermal growth factor receptor.

Supplementary Table S2. Silencer select Validated/Predesigned siRNA and related TaqMan assays.

Gene	Silencer® Select	Taqman gene expression assay
EGFR	siRNA ID #s565<br>siRNA ID #s564	Hs01076090_m1 (# 4331182)
YAP1	siRNA ID #s20367<br>siRNA ID #s20368	Hs00902712_g1 (# 4331182)
NT	siRNA ID #4390843	

EGFR = epidermal growth factor receptor; YAP1 = yes associated protein 1; NT = non-target control.

Supplementary Table S3. TaqMan Gene Expression assays.

**Target**	**Assay**
CTGF/CCN2	Hs00170014_m1 (#4331182)
WTN5A	Hs00998537_m1 (#4331182)
EMP2	Hs00171315_m1 (#4331182)

CTGF/CCN2 = connective tissue growth factor/cellular communication network factor 2; *WTN5A*= wingless-type MMTV integration site family member 5A; *EMP2* = epithelial membrane protein 2.

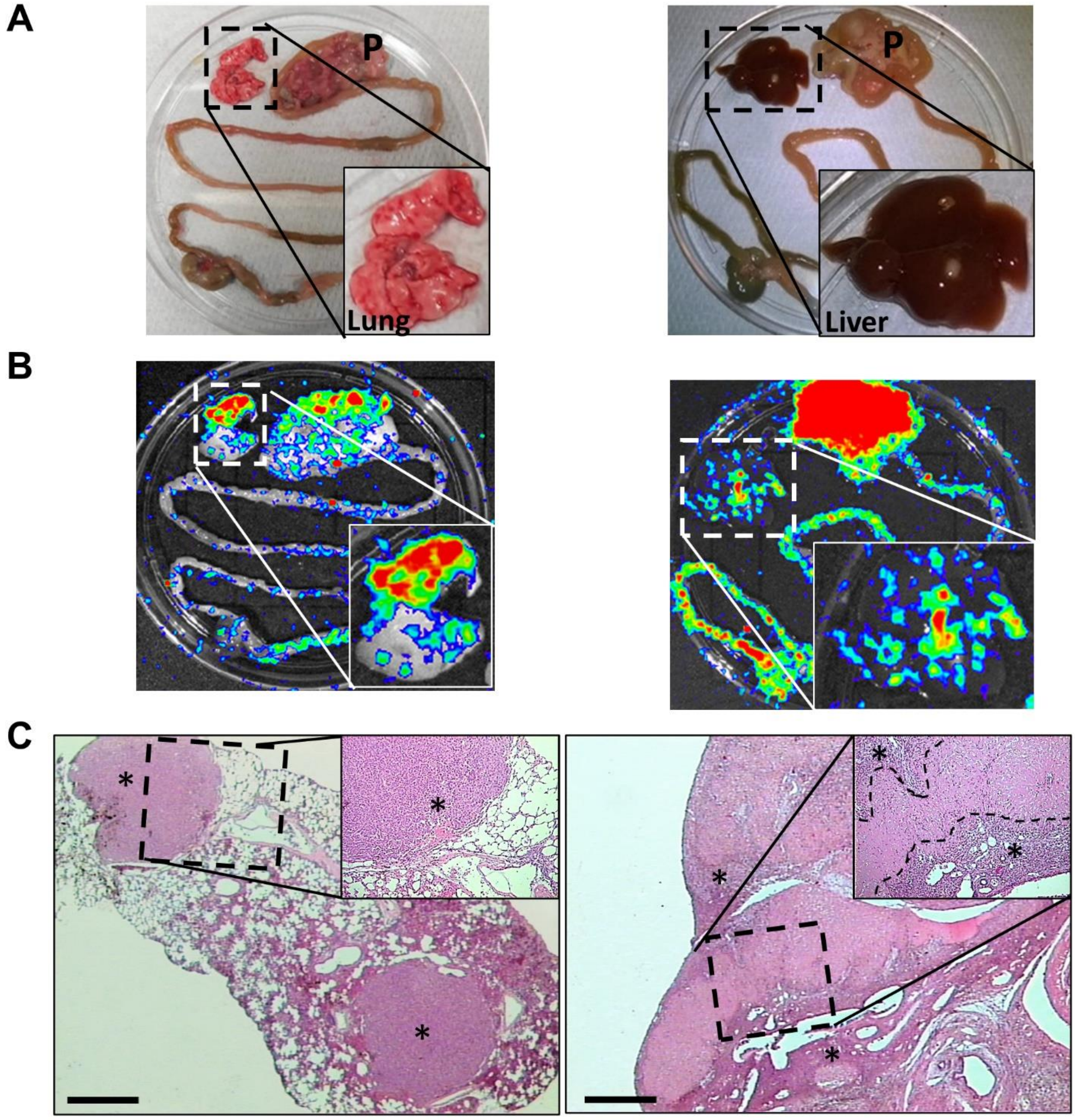

Supplementary Figure S1. Color digital photo (A), *ex vivo* BLI (B) and histological analysis (C) of the lung (left) and the liver (right) of a Diab KCM mice with metastatic PaC. P = pancreas; * indicates metastasis. Original magnification: 25X (in the inset 100X). Scale bar = 500µm.

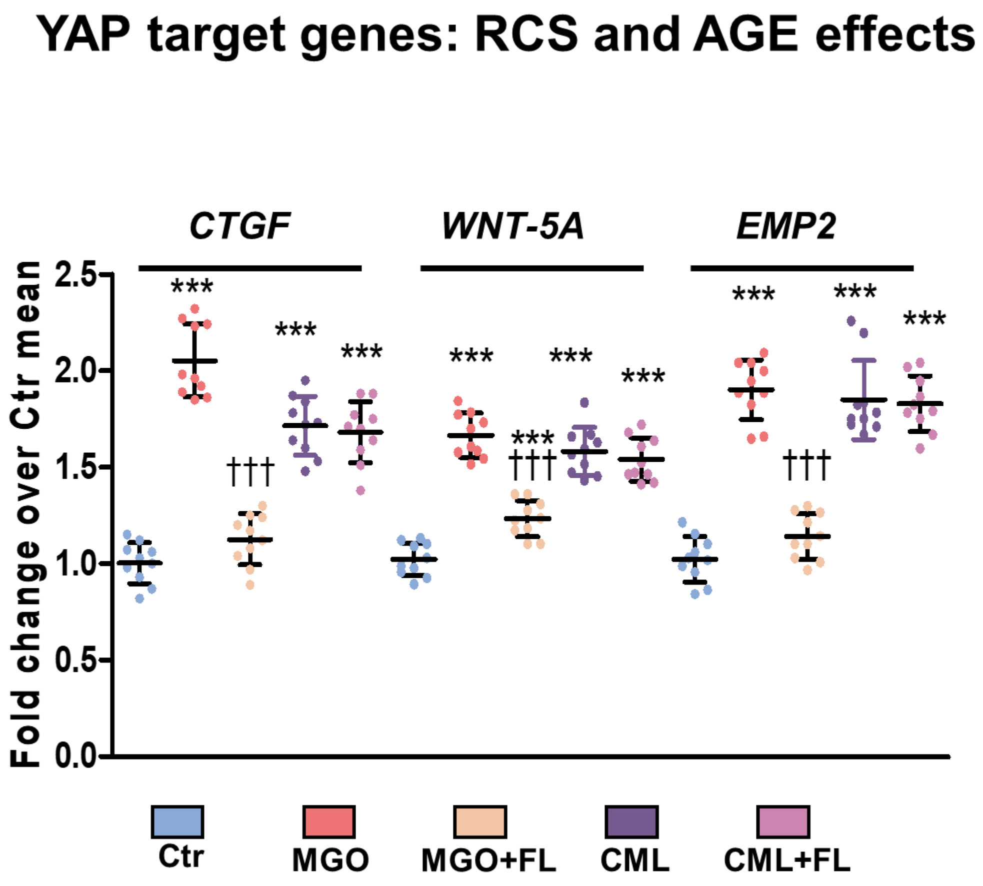

Supplementary Fig. S2. Effect of RCS, AGE and FL-926-16 on YAP activity. *CTGF*, *WNT5a* and *EMP2* mRNA levels (E) in MIA PaCa-2 cells exposed to MGO (200 µM) or CML (100 µg/mL), with or without 20 mM FL; n = 5 wells in duplicate per condition. Each dot represents one well and bars represent mean±SEM. *Post hoc* multiple comparison: ***P< 0.001 vs Ctr; †††P< 0.001 vs untreated.

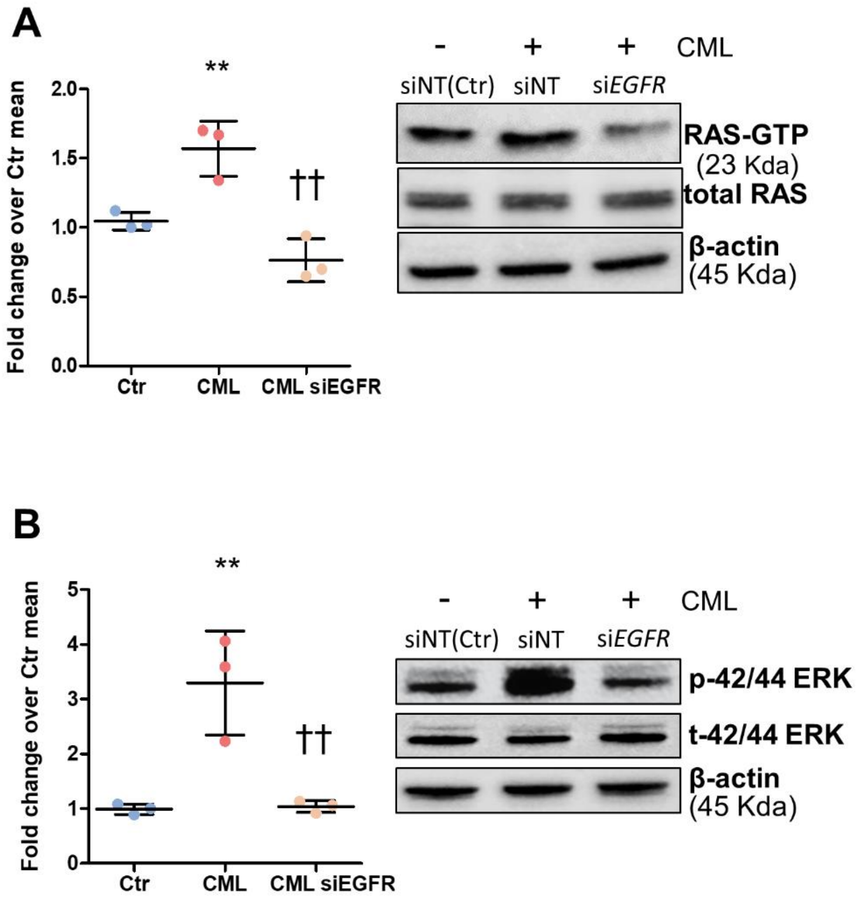

Supplementary Fig. S3. Effect of EGFR silencing on KRAS activity and p-ERK 1/2 levels in human PDA cells exposed to CML. KRAS activity (A), and ERK 1/2 levels (total and phosphorylated) in lysates (B) from human PDA (MIA PaCa-2) siNT control and siEGFR cells exposed to CML (100 µg/mL) for 48 h. Each dot represents a single experiment and bars represent mean±SEM. *Post hoc* multiple comparison: **P< 0.001 vs Ctr; ††P< 0.01 vs CML siNT. siNT = non-target control.
